# Light-weight reference-based compression of FASTQ data

**DOI:** 10.1186/s12859-015-0628-7

**Published:** 2015-06-09

**Authors:** Yongpeng Zhang, Linsen Li, Yanli Yang, Xiao Yang, Shan He, Zexuan Zhu

**Affiliations:** College of Computer Science and Software Engineering, Shenzhen University, Shenzhen, 518060 China; The Broad Institute, Cambridge, MA 02142 USA; School of Computer Science, University of Birmingham, Birmingham, B15 2TT UK

## Abstract

**Background:**

The exponential growth of next generation sequencing (NGS) data has posed big challenges to data storage, management and archive. Data compression is one of the effective solutions, where reference-based compression strategies can typically achieve superior compression ratios compared to the ones not relying on any reference.

**Results:**

This paper presents a lossless light-weight reference-based compression algorithm namely LW-FQZip to compress FASTQ data. The three components of any given input, i.e., metadata, short reads and quality score strings, are first parsed into three data streams in which the redundancy information are identified and eliminated independently. Particularly, well-designed incremental and run-length-limited encoding schemes are utilized to compress the metadata and quality score streams, respectively. To handle the short reads, LW-FQZip uses a novel light-weight mapping model to fast map them against external reference sequence(s) and produce concise alignment results for storage. The three processed data streams are then packed together with some general purpose compression algorithms like LZMA. LW-FQZip was evaluated on eight real-world NGS data sets and achieved compression ratios in the range of 0.111-0.201. This is comparable or superior to other state-of-the-art lossless NGS data compression algorithms.

**Conclusions:**

LW-FQZip is a program that enables efficient lossless FASTQ data compression. It contributes to the state of art applications for NGS data storage and transmission. LW-FQZip is freely available online at: http://csse.szu.edu.cn/staff/zhuzx/LWFQZip.

## Background

The advance of next generation sequencing (NGS) has greatly promoted the research on genomics analysis, hereditary disease diagnosis, food security, etc. The exponential growth of big NGS data outpaces the increase of storage capacity and network bandwidth, posing great challenges to data storage and transmission [1, 2]. Efficient compression methods of NGS data are needed to alleviate the problems [3, 4].

General-purpose compression methods such as gzip (http://www.gzip.org/) and bzip2 (http://www.bzip.org) do not take into account the biological characteristics of DNA sequencing data like small alphabet size, long repeat fragments and palindromes. They fail to obtain satisfying compression performance on NGS data. Accordingly, many specific compression methods have been proposed, the majority of which were designed to process raw NGS data in FASTQ format [5–9] and/or alignment data in SAM/BAM format [10–14] . Depending on whether external reference sequences are used, these specifically-designed methods are widely categorized into two groups namely reference-free and reference-based methods [3].

Reference-free methods, more applicable to FASTQ data, directly store the target sequencing reads with specific compressive encoding scheme based on the inherent statistical and biological nature of the data. For instance, SCALCE [8] clusters the input reads of FASTQ data into groups sharing common ‘core’ substrings using locally consistent parsing algorithm [15], and then compresses each group with gzip or LZ77-like methods. Fqzcomp [7] predicts the nucleotide sequences in FASTQ format via an order-k context model and encodes the prediction results with arithmetic coder. DSRC algorithm [5] divides input reads in an FASTQ file into blocks and compresses them independently with LZ77 and Huffman encoding schemes. DSRC 2 [16] improves DSRC mainly in terms of processing time by introducing threaded parallelism and more efficient encoding schemes. SeqDB [17] compresses FASTQ data of fixed-length reads using block storage like DSRC/DSRC2 together with a data-parallel byte-packing method, which interleaves sequence data and quality scores.

Reference-based methods, by contrast, do not encode the original read data but instead the alignment results, e.g., aligned positions and mismatches, of the reads against some external reference sequences. They are mainly targeted at SAM/BAM data where the alignment results of short reads are readily available. For example, CRAM [12], taking BAM-based input, encodes the differences between reads and a reference genome using Huffman coding. CRAM also includes the idea of using a de Bruijn graph [18] based assembly approach to build an additional reference for the compression of unmapped reads. NGC [10] traverses each column of read alignment in SAM format to exploit the common features of reads mapped to the same genomic positions and stores the mapped reads with per-column run-length encoding scheme. Samcomp [7] makes full use of the SAM flag, mapping position and CIGAR string in a SAM file to anchor each base to a reference coordinate and then uses per-coordinate model to encode the bases. HUGO [11] introduces a specific compression scheme to store the aligned reads in SAM format. The inexact mapped or unmapped reads are split into shorter sub-sequences and realigned against different reference genomes until they are all mapped. Quip [6] implements a standard reference-based compression of SAM/BAM data. It also is equipped with a de Bruijn graph based de novo assembly component to generate references from the target data itself instead of relying on external references, which enables the reference-based compression of FASTQ data.

Reference-based methods tend to obtain superior compression ratios compared to the reference-free ones [3]. Nevertheless, reference-based compression relies on read mapping tools like Bowtie [19, 20], BWA [21] and SOAP [22] to obtain the alignment results against the reference sequence(s). It is noted that these mapping tools are originally designed for other downstream analyses but not for data compression. They tend to involve unnecessary processes and generate undesirable information for the storage of the data. To solve this problem, we propose a novel light-weight mapping model based on *k*-mer indexing strategy to allow a fast alignment of short reads against reference genome(s) and efficiently produce the very essential alignment results for storage.

Based on the light-weight mapping model, a new lossless reference-based compression method namely LW-FQZip is put forward for FASTQ data. Unlike most of the existing reference-based methods that aim at SAM/BAM format, LW-FQZip processes raw NGS data in FASTQ format, as such no extra information brought by SAM/BAM generators is involved in the archive and the maximal compatibility is ensured to the downstream applications. Particularly, LW-FQZip first splits the input FASTQ data into three streams of metadata, short reads and quality scores, and then eliminates their redundancy independently according to their own characteristics. The metadata and quality scores are compacted with incremental and run-length-limited encoding schemes, respectively. The short reads are stored using a reference-based compression scheme based on the light-weight mapping model. Afterward, the three processed data streams are packed together with general purpose compression algorithms like LZMA (http://www.7-zip.org/sdk.html). LW-FQZip was evaluated using eight real-world NGS data sets and the experimental results show that LW-FQZip obtains comparable or superior compression ratios to other state-of-the-art lossless FASTQ data compression algorithms.

The remainder of this paper is organized as follows: Section II describes the framework and implementation details of LW-FQZip. Section III presents the experimental results of LW-FQZip on eight raw NGS data sets. Finally, Section IV concludes this study.

## Methods

### The general framework of LW-FQZip

FASTQ is the most widely used text-based format for storing raw NGS data. An FASTQ file normally contains millions of NGS records each of which consists of four lines. The first line is the metadata containing a sequence identifier and other optional descriptions. The second line is the raw nucleotide sequence, i.e., short read. The third line is typically unused in the downstream analysis and hence can be omitted for compression. The fourth line, of equal length to the second line, is the quality score string with each score reflecting the probability of the corresponding base been correctly determined during sequencing.

**Fig. 1:**
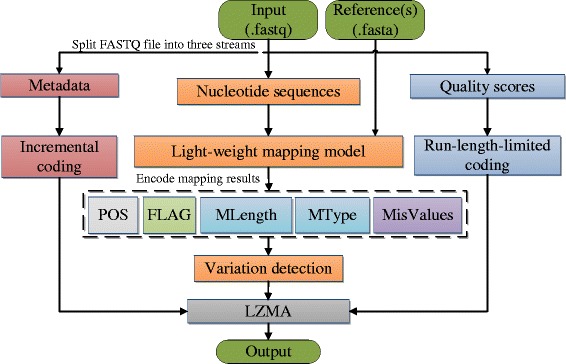
The general framework of LW-FQZip

LW-FQZip follows the framework shown in Fig. 1 to compress an input FASTQ data. It first splits the data into metadata, short reads and quality scores, respectively and then processes them independently with different schemes: the metadata goes through incremental encoding to identify and eliminate repeat segments across different records; the short reads are fed to a light-weight mapping model where they are aligned against an external reference genome and the alignment results are then recorded using a specific encoding scheme; and the quality scores undergo a run-length-limited encoding scheme [23, 24] to abridge consecutive repeats. Finally, a general purpose compression algorithm LZMA is utilized to pack the outputs of the three streams. The details of the compression of the three streams are provided as follows.

### Incremental encoding of metadata

The metadata in each NGS record begins with a symbol ‘@’ followed by a sequence identifier and other optional information like instrument name, flow cell identifier, tile coordinates, and read length. The major part of the metadata is identical for each record, so only one plain copy is needed if the variances in each record can be reserved. In LW-FQZip, the metadata is parsed into different fields and an incremental encoding scheme is used to record the data.

For example, the first four metadata lines of a FASTQ data SRR001471, a NGS data of Homo sapiens generated by Roche/454 GS FLX platform, are shown in Table 1. The first field of a metadata line, i.e., ‘@SRR001471.x’, represents the sequence identifier that is common for all records, so only one copy is needed in storage. The third field, i.e., ‘length = xxx’ indicates the read length and can be omitted in the compression, as the length can be easily acquired from the corresponding short read. The most informative part lies in the second field, e.g., ‘E96DJWM01D47CS’ in the first line, where an incremental encoding scheme is used to record the variances of one metadata to its previous neighbour. Particularly, the first metadata is plainly encoded as ‘SRR001471.1 E96DJWM01D47CS’. The second one is then compared to the first one in the second field to find out nine consistent characters ‘E96DJWM01’ followed by five variances, i.e., ‘CO1KR’, so the second metadata can be encoded with ‘9 CO1KR’, where the length of consistency and the content of variances are delimited with a white space. Similarly, the third and fourth metadata lines are incrementally encoded with respect to the second and third ones respectively.

**Table 1:** Examples of metadata encoding

Original metadata	Incremental coding
SRR001471.1 E96DJWM01D47CS length = 110	SRR001471.1 E96DJWM01D47CS
SRR001471.2 E96DJWM01CO1KR length = 297	9 CO1KR
SRR001471.3 E96DJWM01AL88Q length = 270	9 AL88Q
SRR001471.4 E96DJWM01ALL6A length = 274	11 L6A

### Reference-based compression of short reads based on a light-weight mapping model

It is well known that homogenous genomic sequences share high similarity. When a large portion of the target genomic sequence has been captured by an existing homogenous sequence, i.e., the reference, it is of great benefit to store the target one by recording the differences of it to the given reference [25]. Motivated by this, LW-FQZip aligns the short reads in FASTQ data against an external reference sequence, normally a genomic sequence from homologous species, with an efficient light-weight mapping model and then the alignment results are recorded instead of the original reads.

**Fig. 2:**
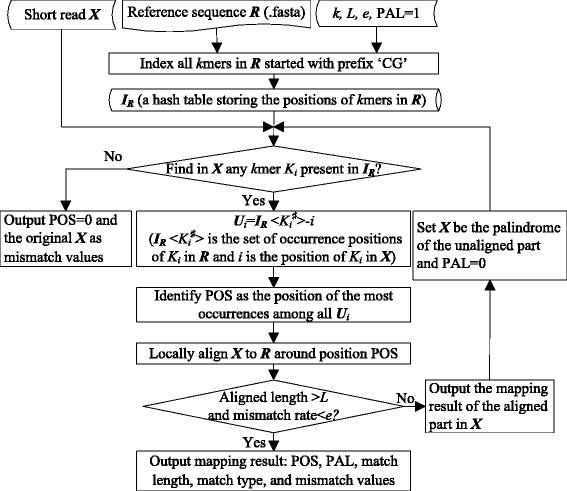
Flowchart of the light-weight mapping model

As shown in Fig. 2, the light-weight mapping model implements fast alignment by indexing the *k*mers within the reference sequence. Firstly, given a reference sequence denoted as ***R***, an index table ***I***_***R***_ is established to store the positions of the *k*mers prefixed by ‘CG’ in ***R. I***_***R***_ is a hash table of numeric keys, which are calculated with a hash function *Hashfunc*(•) taking input *k*mers. Particularly, *Hashfunc*(•) converts a *k*mer say ‘*R*_*i*_*R*_*i+*1_*,*…, *R*_*i+k*_’ to a binary number with ‘A’ = 00, ‘C’ = 01, ‘G’ = 10, and ‘T’ = 11. For example, *Hashfunc*(‘CGATT’) = 0110001111. The value associated with each key, denoted as ***I***_***R***_ < key>, is a set of occurrence positions of the corresponding *k*mer in ***R***. To construct ***I***_***R***_, the program sequentially scans ***R*** and at each time a *k*mer *K*_i_ = ‘*R*_*i*_*R*_*i+*1_*,*…,*R*_*i+k*_’(*R*_*i*_*R*_*i+*1_ = ‘CG’) is detected, a key *K*_*i*_^♯^ is obtained with *K*_*i*_^♯^ = *Hashfunc*(*K*_*i*_) and ***I***_***R***_ < *K*_*i*_^♯^ > is updated with ***I***_***R***_ < *K*_*i*_^♯^ > =***I***_***R***_ < *K*_*i*_^♯^> ∪*i*. The *k*mers serve as seeds for mapping short reads to ***R***. Note that only the most commonly occurring dimer ‘CG’ is considered as the prefix for the sake of speed and memory consumption.

Secondly, the mapping of a short read ***X*** proceeds by identifying in ***X*** any *k*mer *K*_*i*_ that is present in ***I***_***R***_. If there is no *k*mer found in ***X***, the original ***X*** is output as mismatch values with mapped position POS = 0. Otherwise, the set of occurring positions of a *k*mer *K*_*i*_ in ***R***, i.e., ***I***_***R***_ < *K*_*i*_^♯^>, can be retrieved from ***I***_***R***_. The assumed mapped positions of ***X*** on ***R*** using *K*_*i*_ as seed are then represented as ***U***_*i*_ = ***I***_***R***_ < *K*_*i*_^♯^ > −*i*, where *i* is the position of *K*_*i*_ in ***X***. The most frequently occurring position in ***U***_*i*_ is the most likely mapped position of ***X*** on ***R*** (denoted as POS) and a local alignment is thereby performed base by base to find out the exact mapping results. In case there are multiple positions of the same maximal occurrences, local alignments are performed at all positions and POS is set to the one with best matching. Once a valid alignment is found, i.e., the match length is larger than a predefined threshold *L* and the mismatch rate is below *e*, the model outputs the mapping results of ***X***. If the match length is shorter than *L*, the model outputs the mapping results of the aligned part and let the palindrome of the remaining unaligned part go through the mapping model again.

The output mapping results of a read include the mapped position, palindrome flag, match length, match type and mismatch values. We format the results as ‘[POS] < PAL > [MLength] <MType > <MisValues>’. The mapped position POS and match length MLength are mandatory whereas the other fields are optional. Flag PAL is set to 0 if palindrome is used otherwise it is omitted. MLength denotes the number of bases matched/mismatched in the alignment. Whether or not it is a match is indicated in the following field MType that takes one value of ***M*** (Match), ***I*** (Insertion), ***D*** (Deletion), and ***S*** (Substitution). If mismatches are identified, the mismatch values, i.e., one or multiple bases in {‘A’,‘C’,‘G’,‘T’}, should be recorded in the last field MisValues. To boost the matching rate, the mapping model allows a small of number of approximate matches in each alignment. Hence, the read must be reconstructed from the mapping results and compared with the original input to identify the variations. In this way, a lossless compression is guaranteed. If a variation is detected, the position ‘[MisPOS]’ and mismatch values ‘[MisValues]’ are recorded with delta encoding scheme. The descriptions of the output fields are summarized in Table 2.

**Table 2:** The descriptions of the output mapping result fields

Field	Description
POS	The position on reference where the read is optimally aligned.
PAL	PAL=’0’ indicates the alignment of palindrome structure.
MLength	The number of matched or mismatched characters in the alignment.
MType	*M* (Match), *I* (Insertion), *D* (Deletion), or *S* (Substitution)
MisValues	One or multiple bases in {‘A’, ‘C’, ‘G’, ‘T’, ‘N’}

**Fig. 3:**
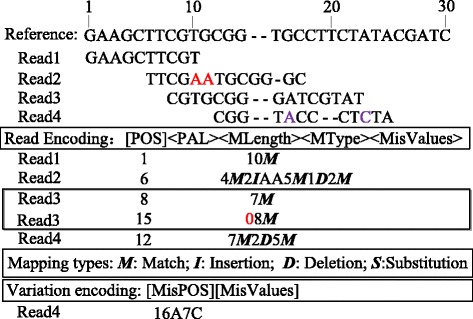
Examples of short read encoding

Examples of short read encoding based on mapping results are shown in Fig. 3. The first short read Read1 is exactly mapped to the reference at the first base, so POS is set to 1 with 10 matches ‘10 *M*’. Read2 is mapped to position 6 with four matches ‘4 ***M***’, followed by two insertions ‘2***I***’ of ‘AA’, four matches ‘4 ***M***’, one deletion ‘1***D***’, and the other two matches ‘2 ***M***’. Read3 is mapped to position 8 of reference for 7 bases but the remainders are unmatched. In this case, the aligned segment is first output as ‘8 7 ***M***’, and the palindrome of the unaligned part is realigned against the reference to find out 8 matches from position 15, so ‘15 08 ***M***’ with a palindrome flag inserted before ‘8 ***M***’ is recorded. Read4 is encoded as’12 7***M***2***D***5***M***’ where two approximate matches in positions 16 and 23 are detected, so an additional delta code ‘16A7C’ is appended at the end.

### Compression of quality scores based on run-length-limited encoding

A quality score *Q* is defined as a property logarithmically related to the base-calling error probabilities *P*, e.g., *Q* = −10*log*_10_^*P*^. Normally, *Q* is quantized and represented with an 8-bit ASCII printable code in FASTQ format. The much larger alphabet size and pseudorandom distribution of quality scores make them harder to compress than short reads [13, 26]. Both lossless [26, 27] and lossy [10, 28, 29] strategies have been explored in the compression of quality scores. In LW-FQZip, only lossless compression is considered to guarantee the fidelity of the data.

The quality scores in FASTQ data usually contain a large number of consecutive occurrences (or called runs) of the same character making them suit for run-length encoding. Particularly, a run-length-limited (RLL) encoding scheme [23, 24] is used in LW-FQZip to record any run of length *n* > 2. For example, a quality score string like ‘CCCGFFFFFFFHH’ can be encoded as ‘C3_α_GF7_α_HH’, where the runs of ‘C’ and ‘F’ are represented with ‘C3_α_’ and ‘F7_α_’, respectively, and ‘G’ and ‘HH’ are plainly recorded. The symbol ‘C’ (‘F’) denotes the repeated character, i.e., ‘C’ (‘F’), by flipping the 8-th bit from the original 0 to 1 so that it is distinguishable from the plainly encoded characters. The length of a run, e.g., ‘3_α_’ and ‘7_α_’, is also represented in an 8-bit ASCII code (unnecessarily printable) to avoid confusion in decoding. As a result, the maximum length of a run is limited to 2^8^ = 256 and any run of length *n* > 256 must be treated as multiple runs.

If consecutive runs appear to be alternate repeats of two characters like ‘DDDDCCCDDDDDCCCC’, the corresponding RLL code ‘D4_α_C3_α_D5_α_C4_α_’ can be further simplified to ‘D4_α_3_α_5_α_C4_α_’, where the solitary ‘3_α_’ indicates the insertion length of the second character recorded in the end of the code. In this way, the coding is shortened from 8*8 bits to 6*8 bits.

As shown in Fig. 1, the separately encoded metadata, short read alignment results, and quality scores are finally packed together and compressed with LZMA.

## Results and discussion

Eight real-world FASTQ data sets ranging from 0.72 GB to 9.88 GB are used to test the performance of LW-FQZip. The data sets were all downloaded from the Sequence Read Archive of the National Centre for Biotechnology Information (NCBI) [30]. The descriptions of the data sets are summarized in Table 3.

**Table 3:** Real-world FASTQ data sets used for performance evaluation

Data	Species	Read Length	Number of Reads	Size (GB)	Reference
ERR231645	*E. coli*	51	6,344,039	1.41	NC_000913
ERR005143	*P.syringae*	2*72	3,551,133	0.89	NC_007005
SRR352384	*S. cerevisiae*	2*76	26,030,832	9.88	NC_001136.10
SRR801793	*L. pneumophila*	2*100	5,406,461	2.75	NC_018140
SRR554369	*Pseudomonas*	2*200	1,657,871	0.82	KI517354
ERR654984	*E. coli*	64-502	1,167,295	1.21	NC_000913
ERR233152	*P. aeruginosa*	77	2,745,192	0.72	AP014622
SRR327342	*S. cerevisiae*	138	15,036,699	5.74	ACFL01000033

The other four state-of-the-art lossless FASTQ data compression algorithms namely Quip [6], DSRC [13], DSRC2 [16] and Fqzcomp [7] are selected for comparison study. Quip [6] is run in two different modes, i.e., the pure statistical compression (‘Quip’) and the assembly-based compression (‘Quip -a’). The general purpose compression algorithm bzip2 is also involved in the comparison as the baseline. All compared methods are tested on a cluster running 64-bit Red Hat Linux operating system with 32-core 3.1GHz Intel(R) Xeon(R) CPU. The parameters of LW-FQZip are empirically set to *k* = 10, *e* = 0.05, and *L* = 12 (The effects of these parameters on the performance of LW-FQZip are investigated and reported in the supplement materials at http://csse.szu.edu.cn/staff/zhuzx/LWFQZip/#Experiment).

The compression ratios of all compared methods on the eight data sets are summarized in Table 4. A compression ratio is obtained by dividing the compressed file size by the original file size. It is observed that all specially-designed FASTQ data compression methods outperform the general purpose method bzip2. LW-FQZip manages to obtain the smallest compression ratios on six out of the eight data sets. Especially, on data ERR654984 of variable-length short reads, the superiority of LW-FQZip is much more obvious. ‘Quip –a’ wins on two data sets namely SRR327342 and ERR231645. It is found that these two data sets contain fewer consecutive quality score repeats than other data sets, so LW-FQZip with run-length encoding fails to obtain comparable compression ratios of quality scores to ‘Quip –a’ that uses a more efficient arithmetic encoding scheme. On average, LW-FQZip obtains the best compression ratio of 0.148 over all datasets, resulting in 85.2 % reduction of the storage space.

**Table 4:** Compression ratios of the compared methods on the eight FASTQ data sets

	quip	quip -a	DSRC	DSRC2	Fqzcomp	LW-FQZip	bzip2
ERR231645	0.139	**0.123**	0.164	0.160	0.136	0.127	0.208
ERR005143	0.154	0.154	0.179	0.176	0.156	**0.151**	0.211
SRR352384	0.115	0.115	0.145	0.144	0.126	**0.111**	0.183
SRR801793	0.184	0.184	0.235	0.234	0.202	**0.176**	0.268
SRR554369	0.194	0.194	0.243	0.232	0.201	**0.182**	0.262
ERR654984	0.188	0.188	0.235	0.236	0.204	**0.140**	0.262
ERR233152	0.129	0.128	0.153	0.147	0.128	**0.126**	0.177
SRR327342	**0.189**	**0.189**	0.242	0.241	0.202	0.201	0.271
Average	0.151	0.150	0.190	0.189	0.162	**0.148**	0.223

The compression ratios of LW-FQZip on the three components of FASTQ data are reported in Table 5. The metadata is best compressed with the smallest average compression ratio 0.021. The compression ratios of quality scores are greater than the other two components due to the inherent larger alphabet size and quasi-random distribution. The compression of quality scores remains the major challenge and opportunity to achieve substantial reduction on storage space of NGS data. Lossy compression could be considered if the loss of accuracy in downstream analyses is controllable.

**Table 5:** The compression ratios of LW-FQZip on the three components of FASTQ files

Data	Metadata	Nucleotide sequence	Quality scores
ERR231645	0.027	0.091	0.421
ERR005143	0.021	0.159	0.364
SRR352384	0.024	0.151	0.130
SRR801793	0.025	0.089	0.371
SRR554369	0.029	0.117	0.346
ERR654984	0.024	0.032	0.285
ERR233152	0.015	0.190	0.238
SRR327342	0.014	0.155	0.426
Average	0.021	0.134	0.268

LW-FQZip is characterized with the light-weight mapping model. The framework can also work with other short read mapping tools like BWA as has been experimented in our previous work [31]. To evaluate the efficiency of the proposed light-weight mapping model, the mapping results, mapping time, and compression ratios using BWA and the light-weight mapping model are tabulated and compared in Tables 6 and 7. With comparable compression ratios, the proposed light-weight mapping model significantly outperforms BWA in terms of mapping time. The light-weight mapping model is much more efficient to obtain the essential alignment results for the purpose of storage. More complete running-time results of LW-FQZip on the test data sets are reported at http://csse.szu.edu.cn/staff/zhuzx/LWFQZip/#Experiment.

**Table 6:** The mapping result of the proposed light-weight mapping model against that of BWA

Data	BWA			Light-weight mapping model			
	#Unmapped reads	#Mapped reads		#Unmapped reads	#Mapped reads		N^a^
		Exact	Inexact		Exact	Inexact	
ERR231645	133,518	6,047,074	163,447	606,902	5,460,749	276,388	17,866
ERR005143	248,883	2	3,302,248	188,801	2	3,362,330	1,417,824
SRR352384	22,182,981	2	3,847,849	22,437,492	1	3,593,339	960,640
SRR801793	424,130	0	4,982,331	1,345,629	0	4,060,832	1,220,888
SRR554369	1,114,495	0	543,376	319,275	0	1,338,596	596,529
ERR654984	3596	0	1,163,699	4403	0	1,162,892	1,107,540
ERR233152	1,005,747	6603	1,732,842	524,812	7375	2,213,005	69,929
SRR327342	14,769,970	0	266,729	14,776,503	0	260,196	64,765
Average	64.39 %	9.77 %	25.84 %	64.91 %	8.83 %	26.26 %	33.54 %

N^*^: the number of unmapped segments that can be realigned to the same reference genome

**Table 7:** Mapping time and compression ratios using BWA and the light-weight mapping model

Data	BWA		Light-weight mapping model	
	Mapping time (s)	Compression ratios	Mapping time (s)	Compression ratios
ERR231645	130.30	0.124	46.60	0.127
ERR005143	533.72	0.150	56.21	0.151
SRR352384	4036.98	0.111	289.02	0.111
SRR801793	1907.43	0.174	79.19	0.176
SRR554369	449.68	0.179	105.33	0.182
ERR654984	282.84	0.146	69.37	0.140
ERR233152	209.38	0.120	82.43	0.126
SRR327342	2458.69	0.201	148.38	0.201
Average	1251.13	0.147	109.57	0.148

## Conclusions

The advance of NGS technologies produces unprecedented data volume and poses big challenges for data storage and transmission. To mitigate the problems, we develop LW-FQZip as a strictly lossless reference-based compression algorithm for raw NGS data in FASTQ format. LW-FQZip compresses the metadata, sequence short reads, and quality scores in FASTQ files based on incremental encoding, reference-based compression, and run-length-limited encoding schemes, respectively. It is characterized with an efficient light-weight mapping model to enable fast and accurate alignment of short reads to a given reference sequence. The experimental results on real-world NGS data sets demonstrate the superiority of LW-FQZip in terms of compression ratio to the other state-of-the-art FASTQ data compression methods including Quip, DSRC, DSRC2 and Fqzcomp. LW-FQZip is mainly designed to optimize the compression ratio, yet parallelism and more efficient coding schemes can be further introduced to improve its time and space efficiency.
